# Ambient-aware continuous care through semantic context dissemination

**DOI:** 10.1186/1472-6947-14-97

**Published:** 2014-12-04

**Authors:** Femke Ongenae, Jeroen Famaey, Stijn Verstichel, Saar De Zutter, Steven Latré, Ann Ackaert, Piet Verhoeve, Filip De Turck

**Affiliations:** Information Technology Department (INTEC), Ghent University - iMinds, Gaston Crommenlaan 8, 9050 Ghent, Belgium; Televic Healthcare NV, Leo Bekaertlaan 1, 8870 Izegem, Belgium; Department Mathematics and Computer Science, University of Antwerp - iMinds, Middelheimlaan 1, 2020 Antwerp, Belgium; iMinds, Gaston Crommenlaan 8, 9050 Ghent, Belgium

**Keywords:** eHealth, Semantic modelling, Continuous care, Context dissemination, Ontology, Ambient intelligence

## Abstract

**Background:**

The ultimate ambient-intelligent care room contains numerous sensors and devices to monitor the patient, sense and adjust the environment and support the staff. This sensor-based approach results in a large amount of data, which can be processed by current and future applications, e.g., task management and alerting systems. Today, nurses are responsible for coordinating all these applications and supplied information, which reduces the added value and slows down the adoption rate.

The aim of the presented research is the design of a pervasive and scalable framework that is able to optimize continuous care processes by intelligently reasoning on the large amount of heterogeneous care data.

**Methods:**

The developed Ontology-based Care Platform (OCarePlatform) consists of modular components that perform a specific reasoning task. Consequently, they can easily be replicated and distributed. Complex reasoning is achieved by combining the results of different components. To ensure that the components only receive information, which is of interest to them at that time, they are able to dynamically generate and register filter rules with a Semantic Communication Bus (SCB). This SCB semantically filters all the heterogeneous care data according to the registered rules by using a continuous care ontology. The SCB can be distributed and a cache can be employed to ensure scalability.

**Results:**

A prototype implementation is presented consisting of a new-generation nurse call system supported by a localization and a home automation component. The amount of data that is filtered and the performance of the SCB are evaluated by testing the prototype in a living lab. The delay introduced by processing the filter rules is negligible when 10 or fewer rules are registered.

**Conclusions:**

The OCarePlatform allows disseminating relevant care data for the different applications and additionally supports composing complex applications from a set of smaller independent components. This way, the platform significantly reduces the amount of information that needs to be processed by the nurses. The delay resulting from processing the filter rules is linear in the amount of rules. Distributed deployment of the SCB and using a cache allows further improvement of these performance results.

## Background

### Introduction

Since a number of years, the complexity of institutional care settings has been increasing due to societal factors such as the growth of the care unit size, the more specialized nature of the care and the reduction in staffing levels. This requires a more optimized rostering and use of staff resources.

Information technology is often introduced [1] to deal with these issues. The current institutional care settings contain numerous devices and applications to support caregivers in carrying out their everyday activities, e.g., electronic medical records or patient monitoring equipment. However, this high amount of technology further increases the complexity of these daily activities, because the caregivers are directly faced with often complex technologies [2]. The caregiver has to use several devices to consult and insert data even when carrying out a single task. This is very time-consuming. Due to this inadequate integration of the available technology, as well as the large amount of data being generated by the devices and the heavy workload of staff members, it is not uncommon for important events to be missed, e.g., early indications of worsening condition of a patient.

Consider for example a patient with a concussion, who needs to be in a dark environment. Today, the staff members are responsible for switching on the lights at the appropriate level each time they enter the room. Consequently, each staff member has to be aware of all the aspects and specificities of the patient’s condition. If an uninformed person enters the room or a wrong button is pressed, this can cause physical pain for the patient. However, if the lighting control system would be aware of the patient’s pathology and needs, it can automatically turn on the light to the correct level when it detects that the nurse enters the room. Moreover, a message can be displayed explaining to the nurse why the lights are dimmed. Staff members are able to overrule the system and a light sensor could be used to monitor the light intensity in the room and alert a nurse if a pre-defined threshold is crossed.

### Ambient-aware continuous care

The ultimate ambient-intelligent care room of the future uses numerous devices to sense the needs and preferences of the patients and caregivers and adapt itself accordingly [3]. This implies an emerging demand for the integration and processing of the heterogeneous data offered by the different technologies present in the room.

The ACCIO [4] (Ambient-aware provisioning of Continuous Care for Intra-muros Organizations) project aims to realize this goal by developing a context-aware, ambient-intelligent, pervasive and semantic platform. This platform, called the Ontology-based Care Platform (OCarePlatform), enables technology to blend into the background by using sensors to sense the current context and actuators to adapt the environment according to the sensed context [5]. The OCarePlatform integrates, filters and processes the large amounts of heterogeneous care data delivered by the various devices, sensors, applications and clinical databases in order to optimize continuous care processes. This frees the caregiver from the cumbersome task of managing the different sensors, actuators and applications him- or herself. However, to achieve this goal, the platform must be able to interpret the meaning and adequately filter the relevant information out of this huge amount of care data. Unorganized raw data can be voluminous, but has no meaning on itself as it has no relationships or context. Information is data that has been given meaning by defining relational connections. For this, the platform uses an ontology [6], which is a semantic model that formally describes the concepts in a certain domain, their relationships and attributes. By managing the data about the current context in an ontology, intelligent algorithms can be more easily defined that take advantage of this information to automate, optimize and personalize the continuous care of patients. Referring back to the previous example, this means that the ontology models the patient’s condition and the nurses’ locations amongst other things. Algorithms can then be expressed, using concepts from the ontology, to automatically dim the light to the correct level based on, e.g., the location of the patient and the nurses, the time of day and the condition of the patient. Afterward, the nurse can decide to overrule this decision by adjusting the light level in the room manually. This allows that unexpected events can be addressed by the caregiver.

### Objective & paper organization

The main research question addressed in this paper is: How do we build a modular platform that is able to process and intelligently reason on the large amount of heterogeneous care data in a performant an scalable manner in order to optimize continuous care processes?

The remainder of this article is structured as follows. The “Related work” Section highlights the contributions of this paper in view of the relevant related work. The “Methods” Section starts with a description of the architecture of the OCarePlatform in the “Architecture of the OCarePlatform” Subsection. The “Continuous care ontology” Subsection describes the developed continuous care ontology. The “Use case: optimizing continuous care through an ontology-based nurse call system” Subsection elaborates on the specifics of the platform using an illustrative example, namely realizing a next-generation nurse call system supported by a Localization and Home Automation Component. To demonstrate the advantages and evaluate the performance of the OCarePlatform, the prototype was evaluated in a living lab environment. The evaluation set-up is detailed in the final subsection of the “Methods” Section. The “Results and discussion” Section evaluates the amount of data that is filtered by the OCarePlatform and the performance and scalability of the filter rules. It also discusses the potential impact of the platform on the delivery of continuous care. The conclusions are highlighted in the “Conclusions” Section.

## Related work

### Context-aware systems

Dey and Abowd [7] refer to context as “any information that can be used to characterize the situation of entities (i.e., whether a person, place or object) that are considered relevant to the interaction between a user and an application, including the user and the application themselves”. A system may be labeled as “context-aware” if it can acquire, interpret and use context information to adapt its behavior for the current context in use [8]. A number of generic context platforms have been developed previously to relieve application developers from the aggregation and abstraction of context information and the derivation of high-level contexts. A complete overview and classification of the literature can be found in Hong, et al. [9], while an in-depth discussion and comparison of these platforms can be found in Baldauf, et al. [10] and Xue and Pung [11].

One of the first platforms to be developed was the Context Toolkit [12], a Java framework allowing the rapid prototyping of sensor-based context-aware applications. However, the Context Toolkit does not provide a common context model to enable knowledge sharing and context reasoning. Various approaches have been proposed for modeling context information, i.e., key-value, markup scheme, graphical, object-oriented, logic-based and ontology-based models. Strang and Linnhoff-Popien [13] evaluated these context modeling approaches based on six criteria, namely distributed composition, partial validation, richness and quality of information, incompleteness and ambiguity, level of formality and applicability to existing environments. They show that ontologies fulfill most of these requirements and are the most expressive models as these formal models allow the integration and exploitation of more specific context knowledge with high-level context information using reasoner components. The most prominent examples of context-aware systems based on ontologies are the Service-Oriented Context-Aware Middleware (SOCAM) [14], the Context Broker Architecture (CoBrA) [15], the Context Managing Framework (CMF) [16], a service-oriented middleware that integrates a Context Management Service with an Awareness and Notification Service (CMSANS) [17], the service-oriented architecture for context-aware middleware in a smart home described by Kim and Choi [18] and Gaia [19]. The properties of these systems are summarized in Table 1.

**Table 1:** **Comparison of prevalent ontology-based generic and healthcare context-aware systems to the OCarePlatform**

Context-aware systems	Context model	Contex reasoning	Reasoning expressivity
CoBrA	Centralized	Logic & rule-based	RDFS & OWL-Lite
CMF	Centralized	Machine learning	Naive Bayesian classifier
CMSANS	Centralized	Logic & rule-based	RDF
SOA	Centralized	Logic & rule-based	RDF
SOCAM	Hybrid	Logic & rule-based	RDFS, OWL-Lite, Jena Rules, Prolog & hybrid
Gaia	Distributed	Logic & rule-based	DAML + OIL
Fook et al.	Centralized	Logic & rule-based	OWL-DL
Zhang et al	Centralized	Logic & rule-based	OWL-DL, Jena Rules, Prolog & hybrid
ERMHAN	2 nodes	Logic & rule-based	OWL-DL & Jena Rules
OCarePlatform	Distributed	Logic & rule-based	OWL-DL, SWRL & Jena Rules

A distinction can be made between systems that keep the context information centralized or distributed. In a centralized system, one common knowledge component is used, which integrates all the context information and inferences high-level knowledge through reasoning on this shared context model. Various applications can then access this knowledge or query this shared context model. The central context server is also able to monitor context changes and send events to the interested applications. CMF, CMSANS, CoBrA and the SOA proposed by Kim and Choi all use this approach. The disadvantage of the centralized approach is that the context server forms a single point of failure and a performance bottle neck. To avoid the first problem, CoBrA offers the possibility of creating brokered federations. A federation consists of multiple instances of the central knowledge component, called the context broker. These brokers then periodically exchange and synchronize contextual knowledge. An advantage of this approach is that the access to the shared context model no longer depends on the availability of one single broker. Another broker from the federation can easily be used to replace the one that becomes unavailable. However, as each broker contains all the context information, performance remains an issue.

In the distributed approach, the context information is distributed across multiple components. None of the components thus has a complete overview of the current context. GAIA supports both pull and push-based context acquisition. The former is enabled by letting the applications specify queries for specific context information. For the latter, GAIA uses communication channels. Each channel has one or more context suppliers. The applications, called consumers, can register for context information they are interested in.

SOCAM employs a hybrid approach. Applications can directly receive context information from the different context providers. However, the context providers also supply their knowledge to a central knowledge component, called the *Context Interpreter*. This Context Interpreter maintains a shared context model and derives high-level knowledge from it. It offers this knowledge to the different applications. The applications thus receive both low-level as well as high-level context information.

The ontology-based context-aware systems also differ in the used knowledge representation language. The most common language for describing ontologies is the Web Ontology Language (OWL) [20]. Three variants of OWL exist, with different levels of expressiveness, namely OWL-Lite, OWL-DL and OWL-Full (ordered by increasing expressiveness). OWL-Lite allows expressing a classification hierarchy and simple constraints. OWL-DL is founded on Description Logics(DL) [21]. Description Logics are decidable fragments of first-order logic. Consequently, the OWL-DL variant provides the maximum expressiveness possible while retaining computational completeness, decidability and the availability of practical reasoning algorithms. As a result, semantic reasoners, such as Pellet [22] or Hermit [23], can be used to infer new knowledge, i.e., logical consequences, from the information captured in OWL-Lite and OWL-DL ontologies. Rules, e.g., Semantic Web Rule Language (SWRL) [24] or Jena Rules [25], can be expressed using OWL concepts. These rule languages support a wide range of built-in operators which greatly increase expressiveness of the context model. Table 1 summarizes the reasoning capabilities for the aforementioned context-aware platforms.

### Context-awareness in healthcare

Context-aware computing is a research field, which considers healthcare as a relevant area of application [26]. Especially pervasive healthcare is highly suitable for using context-aware systems [27]. First, there is a large amount of available information, specific healthcare situations and related tasks, which creates a potential for cognitive overload amongst the caregivers. Second, the patients, healthcare professionals and some equipment are fairly mobile, which requires accurate localization and adaptation of the healthcare services to the environment. Third, the financial and human resources are limited. This implies a need to cut cost while improving the quality of care to an increased number of people. Although context-awareness infrastructure including more complex devices and software will add to the total cost, the reduced number of medical errors and the ability to more effectively utilize healthcare resources should lead to reduced cost [27]. Finally, the expectation to be able to access, process and modify healthcare information anywhere using mobile devices is another reason to use context-aware approaches.

Context-aware and pervasive technologies have been applied to a number of hospital use cases [26]. The following notable prototypes have been proposed in literature. The “hospital of the future” [28] prototype consists of (1) a context-aware Electronic Patient Record (EPR) filtering information according to the current context, (2) an intelligent pill container for proper dose administration and (3) a context-aware hospital bed of which the content of the display changes according to the context and displays warnings for some potentially incorrect actions. The Context-aware MobileWard [29] is designed to support nurses in conducting morning procedures in a hospital ward. An intelligent hospital prototype [30] has been developed, which allows localization of a team member and the ability to initiate an audio-video conference from the nearest contact point. Similarly, the Vocera communication system [31] supports communication amongst hospital workers via mobile devices and localization techniques. In Muñoz, et al. [32] a context-aware mobile communication prototype is presented, which empowers mobile devices to recognize the context in which hospital workers perform their tasks in order to provide contextual messaging.

Similarly, the following notable context-aware prototypes have been developed for homecare and residential care. Prototypes have been proposed to assist patients in taking their medications at home [33, 34]. Vivago [35] is a social alarm system for elderly based on wearable sensors. It provides long-term monitoring of a user’s activity profile and automatic alarm notification. A context-aware system has also been developed to assist adults with dementia during hand washing [36]. The use of context-aware systems for telemedicine of chronic diseases [37, 38] is also an active research field. The LifeMinder prototype [39] can sense pulse waves, user’s actions and postures and can capture contextual photos and continuous voices. This information is then used to detect that the user is in a stressful state. Finally, a prototype [40] was presented to detect falls of elderly by using a visual fall detection system and combining this with context information, e.g., patient’s general condition, location and duration of patient’s inactivity.

Examples of context-aware healthcare systems based on ontologies can also be found in literature. Fook, et al. [41] present a context-aware system for monitoring and handling agitation behavior in persons with dementia. Zhang, et al. [42] propose a context-aware infrastructure to support the global healthcare system in terms of device access, context management and service interoperability. These two approaches adopt a centralized knowledge management approach with an ontology-based knowledge component as central entity. The Emilia Romagna Mobile Health Assistance Network (ERMHAN) [43] is a multichannel context-aware service platform designed to enable the development and delivery of an extensible set of care services which allow patients to be assisted at home and support the activity and mutual collaboration of care providers who are involved in patient care and assistance. This framework distributes the context knowledge across two nodes. The *Patient Context Manager* is deployed at the patient site and is responsible for pre-processing the data retrieved by biomedical and environmental sensor networks. Rule-based reasoning is employed to detect abnormal phenomena in this sensor data and to forward these anomalies to the *Central Context Manager*. This *Central Context Manager* is deployed in the care center and combines the alarms received from the *Patient Context Manager* with other context information in order to take appropriate actions. The properties of these systems are also summarized in Table 1.

The healthcare scenario has some specific implications which differentiate it from other scenarios. Although much research has been done on the subject, the adoption of context-aware services is lagging behind what could be expected. Most of the aforementioned projects are prototypes. Real applications are still difficult to find. Whereas the healthcare industry is quick to exploit the latest medical technology, they are reluctant adopters of modern health information systems [44]. Half of all computer-based information systems fail due to user resistance and staff interference [45]. The main complaint made against mobile, context-aware systems is that users had to significantly alter workflow patterns to accommodate the system [46]. An often overlooked fact is that the strength of any context-aware platform is heavily dependent on the correctness and completeness of the used knowledge model. This model needs to capture the daily work practices and context of the caregivers and patients accurately [47]. Constructing this model is a difficult task. In contrast to the healthcare domain in general, a lot of knowledge used in continuous care, e.g., how to prioritize and assess nurse calls or assign caregivers to patients, is implicit and best practices are not rigorously documented.

Another challenge in the healthcare scenario is the fact that wrong decisions made by the system can have severe implications. Context data delivered by sensors is very unreliable. Decisions made based on wrong or incomplete sensor data might thus not be correct. Quality of Context-aware (QoC-aware) algorithms that take the reliability and correctness of the context data into account should thus be developed to mediate this issue.

### Publish/subscribe systems

Publish/subscribe systems have evolved from static topic-based to dynamic content-based systems. By augmenting the content with semantics, subscriptions can be created which take into account the actual meaning of the content. Several semantic publish/subscribe systems have been proposed in literature [48], which differ in the method proposed to relate subscriptions to messages, namely based on Resource Description Framework (RDF) [49] graph-matching, ontological inferencing and attribute-value pair matching. Our approach is most closely related to semantic publish/subscribe systems that use OWL inferencing. These systems represent subscriptions as OWL concepts and messages as concept instances. An inferencing engine is used to determine if a message instance satisfies the constraints of a subscription class. This approach is more expressive than the custom RDF graph-matching algorithms as it allows new, non-asserted knowledge to be inferred. Moreover, it does not limit the formatting of the messages, as is the case in systems based on attribute-value pairs.

### Ontologies for representing context in healthcare environments

The definition and use of ontologies in the medical domain is an active research field, as it has been recognized that ontology-based systems can be used to improve the management of complex health systems [50]. However, most of the developed ontologies focus on the biomedical domain and are mainly employed to clearly define medical terminology [51], e.g., Galen Common Reference Model [52] or the Gene Ontology [53].

A very well-known, comprehensive, multilingual clinical healthcare terminology is SNOMED CT [54]. SNOMED CT focuses on defining clinical terminology, providing terms, synonyms, codes and definitions used in clinical documentation, clinical health records and reports. It contains more than 300,000 concepts. However, the focus is more on describing the condition of the patient, his or her clinical information and the procedures he or he has undergone. As such it is tuned a lot towards healthcare provided in hospital settings (focus on curing the patients) and not so much in residential or home care settings (focus on caring for patients). Consequently, it does not represent information and knowledge used to optimize continuous care work processes. It does not describe, e.g., the different care tasks such as making the bed, giving a patient toilet assistance or answering nurse calls and the competences that are required for it, the various roles a caregiver can assume and the competences that are associated with it and the association between measured parameters, the devices that measure them and the faults and alarming measurements that might occur. Moreover, the formalism in which SNOMED CT is expressed is much less expressive than OWL. There are still known deficiencies regarding the ontological commitment of SNOMED CT [55, 56], e.g., the clarification of which kind of entity is an instance of a given SNOMED CT concept. This means that different agents might interpret SNOMED CT differently, depending on their point of view. This means that it is difficult for existing OWL reasoners to reason over and classify the SNOMED CT ontology in an automated and consistent way. Moreover, when all the instance data of different patients is added to this large ontology, it becomes very difficult to reason over it with existing reasoners in a scalable and performant manner. As such, we deem SNOMED CT ideal to integrate and exchange healthcare data in standardized manner, however, it is not ideal to support reasoning over this data to optimize continuous care.

Different standards have also been proposed to support the continuity of care for patients. Continuity of care is concerned with the quality of care over time. As patient’s healthcare needs can rarely be met by a single professional, continuity of care is achieved by providing a seamless service to patient through integration, coordination and the sharing of information between different healthcare providers and caregivers. The most well-known of these standards is Health Leven Seven (HL7), which provides standards for the exchange, integration, sharing, and retrieval of electronic health information. However, these standards suffer from the same drawbacks as previously mentioned for the ontologies, i.e., they focus on clinical information and procedures and do not contain standardization efforts towards continuous care processes, e.g., making the bed or answering patients calls.

Unfortunately, it can be concluded that little work has been done on developing high-level ontologies, which can be used to model context information and knowledge utilized across the various continuous care settings. However, ontologies have been developed for specific subdomains of continuous care, e.g., ontologies for structuring organizational knowledge in homecare assistance [50], representing the context of the activity in which the user is engaged [57] and modeling chronic disease management in homecare settings [43]. The used contextual information is often very simple. Time, location and profile information of staff members and patients are the most used contextual parameters [26]. A major challenge in modeling context-aware healthcare ontologies is that the description of a situation by using what (activity), who (identity), where (location) and when (time) may not be enough [27]. More richness and higher reliability are required. This could include how (process), with whom (sources), and so what (needed action).

### Our contribution

In this research, the OCarePlatform, a distributed, scalable, context-aware and pervasive platform to support continuous care processes, is presented. This platform employs a Semantic Communication Bus (SCB) to accomplish a flexible and semantic publish/subscribe mechanism to communicate context information between the devices delivering context information and the applications processing this information. Table 1 compares the OCarePlatform to the prevalent generic and healthcare ontology-based context-aware systems. It can be noted that our approach adopts a distributed context model. The SCB, which uses a set of core ontologies to model the communicated context information, forwards the gathered context information to the various applications, but does not retain this information. The applications have their own individual knowledge component, which contains a domain-specific extension of (a subset of) the core ontologies. The applications do retain the context information they obtain. To communicate to the SCB in which information they are interested, the applications register filter rules with the SCB. They are also able to post inferred knowledge back to the SCB. The SCB thus loosely couples the context providers and the content consumers, i.e., the applications. None of the components have a complete overview of the current context as the knowledge is distributed across the various applications. In contrast to a shared context model, the application components only manage the context model and context information pertaining to their specific domain. This improves the scalability and performance of these applications, as they need to manage less context information with more concise context models. Consequently, expressive inferencing, i.e., OWL-DL, SWRL and Jena Rules inferencing, can be efficiently performed. It can be derived from Table 1 that the use of a distributed context model has not found a lot of uptake in healthcare context-aware systems. Most systems use a centralized context model. ERMHAN only distributes the context model across two nodes, one at the patient’s home and one in the care center. This distribution is thus location-based. Our approach distributes the context according to application domain.

Additionally, our approach differs from other OWL inferencing publish/subscribe systems as it also allows the use of Jena rules and SWRL [24] to define subscriptions. These rule languages support a wide range of built-in operators which greatly increases expressiveness. Jena [25] rule inferencing exhibits the best scaling behavior in function of the amount of subscription rules and increasing message complexity [58, 59], but is less expressive than SWRL or OWL inferencing. The choice between these three reasoning approaches allows balancing expressivity and performance according to the specific use case at hand. The generic context-aware platform with semantic publish/subscribe mechanism presented in this paper has already been applied to several autonomic network management scenarios such as the management of a multimedia access network and the management of a cloud data center [59]. This paper thoroughly evaluates the performance of the proposed platform within the healthcare domain. Moreover, it is shown how a more scalable platform can be achieved by distributing the SCB and employing a cache.

As mentioned previously, little work has been done on the development of high-level continuous care ontologies, which can be re-used across the various continuous care domains, e.g., hospitals, care residences and homecare. Therefore, seven continuous care core ontologies and a plethora of domain-specific ontologies, which import (a subset of) these core ontologies, were designed. The ontology was developed in such a way that it can easily be extended with new knowledge.

As a last contribution, this paper also presents a framework and algorithms allowing applications to autonomously generate and register new filter rules based on the current context.

In summary, the main contribution of this paper is the presentation of the design of the OCarePlatform, which combines expressive OWL-DL context reasoning, distributed management of the context model and information, intelligent filtering of context information, and distributed deployment of the publish/subscribe mechanism and inclusion of a cache to increase scalability. The combination of all these features differentiates this platform from other works in the same area. This paper also illustrates the flexibility of the OCarePlatform by elaborating on the framework and algorithms that allow the application components to adapt the filter rules and thus the information they are interested in. Finally, this paper also presents the performance evaluation of the SCB in the healthcare domain using the developed continuous care ontology.

## Methods

### Architecture of the OCarePlatform

**Figure 1:**
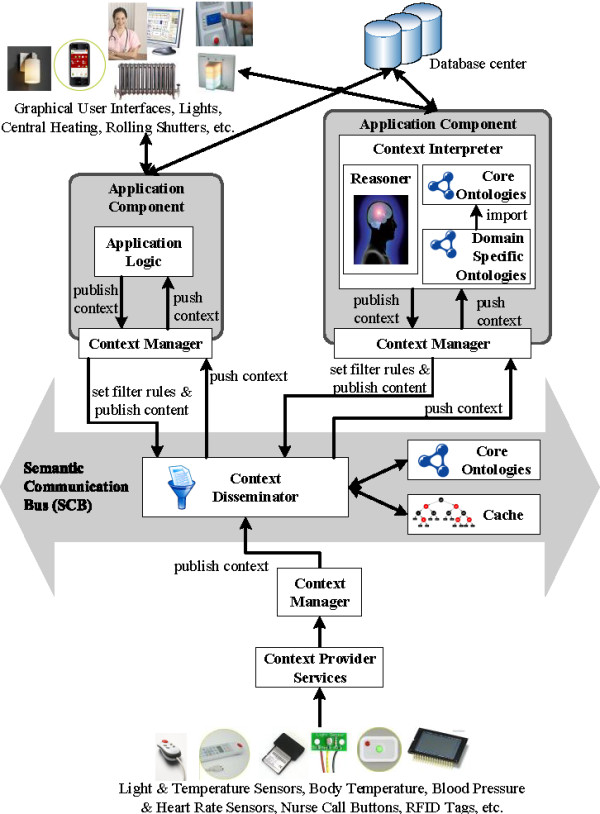
**Architecture of the OCarePlatform using a semantic communication bus for interaction, collaboration and orchestration.**

The ambient-intelligent care room consists of various devices, e.g., sensors and nurse call buttons, and intelligent applications that process the generated data. A communication substrate is needed to glue these components together and orchestrate collaboration. For this, the *Semantic Communication Bus (SCB)*[59] was designed, as visualized in Figure 1. The SCB orchestrates the communication of semantically enriched data. This allows filtering data based on meaning rather than on string patterns. The SCB interprets the data by using *core ontologies* which model the information being exchanged for a continuous care domain. For example, the ontologies represent that the environment contains light sensors, which make observations about the light intensity at a location. As mentioned previously, little work has been done on developing ontologies to support the continuous care of patients. Such an ontology has to contain information about the profile of staff members and patients, roles and responsibilities, administrative information, etc. To tackle this issue, a participatory ontology engineering methodology was developed in previous research to co-create together with the stakeholders the continuous care core ontologies. This is further detailed in the “Continuous care ontology” Section.

As depicted at the bottom of Figure 1, the *Context Provider Services* receive data from the devices in the environment and transform it to context information which adheres to concepts in the core ontologies. This semantically enriched information is forwarded to the *Context Manager*, which publishes it on the SCB. For example, the *Location Provider Service* is used to publish location information about staff members or patients on the SCB.

As is the case with classic publish/subscribe mechanisms, the SCB contains a *Context Disseminator*, which allows application components to subscribe to context information, which is relevant to them at that specific moment. The application components use a *Context Manager*, which contains (a subset of) the core ontologies used by the SCB, to specify the context they are interested in. This is done by defining *filtering rules* and registering them with the *Context Disseminator*. For example, a nurse alerting application component indicates that it is only interested in light intensity observations, crossing a particular threshold and coming from rooms with patients who suffer from a concussion. These rules are expressed using concepts from the core ontologies. Examples of such rules can be found in the “Flexible and semantic publish/subscribe mechanism” Section. The application components can also register new filter rules on the fly based on the current context, which greatly improves the flexibility of the platform. This is further detailed in the “Flexible and semantic publish/subscribe mechanism” Section.

When continuous care information is published on the SCB, the *Context Disseminator* matches this published event with the registered filter rules by reasoning on the information in the ontology. If a match is found, the information is forwarded to the application components that subscribed to that filter rule.

As visualized in the top right of Figure 1, the intelligent application components, receiving context information from the SCB, also use ontologies to model their specific (sub)domain and perform sophisticated reasoning. These *domain-specific ontologies* extend concepts from the core ontologies, such that the context delivered by the SCB is directly understood by the application logic. Static information about the environment is collected from databases, e.g., names of patients or locations of sensors. This also allows integrating information collected from the electronic health record (EHR) of the patient in the domain ontologies used by the application components. The electronic health record is checked when a new patient is admitted and the relevant information is loaded into the ontology. During the stay, the electronic record and the ontological knowledge base are also constantly synched. As such, this information is taken into account when reasoning is performed about the current context and condition of the patient.

As a result of the reasoning, the application components can adapt the environment by controlling devices, e.g., lights or alerting a caregiver by sending a notification to a mobile phone or beeper. The application components can also publish their conclusions on the SCB through the *Context Manager*. This way, they can be picked up by other application components to perform additional reasoning. For example, a first application component computes the locations of people, based on the available raw sensor information, and publishes these locations on the SCB. A second application component uses this augmented location information to assign staff members to tasks, while a third application component uses it to regulate the light level in a room. As the different components on which the complex reasoning is based can run in parallel, this allows achieving complex reasoning in a very performant and scalable manner.

Note, that the Semantic Communication Bus (SCB) only reasons about one event at the time. It classifies the event according to its properties and forwards it to the application components that are interested in these types of events. This allows that the SCB can easily be replicated and distributed to increase its performance and scalability. This is further explained in the “Flexible and semantic publish/subscribe mechanism” Section. The application components then reason further on the events they receive from the SCB. This reasoning possibly involves reasoning on previous events and the data recorded about the patient and captured in an EHR or other databases. This allows building very modular application components that perform very specific tasks. Moreover, the use of filter rules reduces the amount of care data that is forwarded to these components. This means that they perform their specific tasks, i.e., reasoning, only on that subset of the generated events that they are interested in. This prevents these components from being flooded with huge amounts of sometimes irrelevant data generated by the sensors and devices in a truly pervasive and ambient-aware patient room. It thus allows increasing their performance.

To ensure that the SCB can process all the events it receives in a timely manner, the Context Disseminator uses a cache [60]. When information is published on the SCB, the Context Dessiminator first checks the cache. If the published event is not found in the cache, a miss occurred and the Context Disseminator performs semantic reasoning to determine to which filter rules the event matches. If a match is found, the event is forwarded and the match is added to the cache. The cache thus contains mappings between published events and the matching filter rules. However, if the published event is found in the cache, a hit occurred. The filter rules to which this event is mapped are collected and the event is forwarded to the application components that subscribed to the filter rule. Consequently, no reasoning needs to be performed to process the event. Efficient search algorithms exist to implement a cache, which allow performing a cache look-up in a very performant manner [61].

### Continuous care ontology

A continuous care ontology was developed using a participatory ontology engineering methodology. The methodology is tuned towards less IT-focused domains, such as healthcare, where the stakeholders might not be willing or able to construct the ontologies themselves. It involves the stakeholders, e.g., nurses, caregivers and physicians, social scientists and ontology engineers in each step of the ontology development cycle by employing several participatory methods and techniques to capture the daily and preferred work practices, e.g., observations, role-playing and discussing scenarios in hands-on workshops. A detailed discussion and evaluation of the methodology can be found in Ongenae, et al. [62, 63].

Knowledge about a certain domain constantly changes such as the discovery of new drugs and diseases. An ontology is therefore not a static model, but a dynamic one that should constantly be able to change and evolve. This was taken into account by developing the continuous care ontology in such a way that the model can easily be extended with new concepts, relations and axioms.

As a first measure, a distinction was made between general and domain-specific continuous care knowledge. The first is of interest to various context-aware healthcare applications and is applicable across all continuous care domains, e.g., hospitals, home care environments and residential care settings. This knowledge is modeled in the continuous care core ontologies. Adding too many axioms to the core ontologies that constrain the possible interpretations of concepts was especially avoided, unless there was very wide agreement about the constraint amongst the stakeholders involved in the co-creation process. This facilitates cross-domain applicability of the core ontologies and allows easy extension without contradicting with the knowledge already contained in these ontologies. The domain-specific ontologies, which were also developed using the participatory ontology engineering methodology, model knowledge particular to a specific domain, e.g., the specific roles and competences employed within a hospital setting and how they map on each other, the specific patient profiles present within a care residence or the particular continuous care workflows pertaining to nurse calls or care requests. All concepts in the domain-specific ontologies are always subclasses of concepts in the core ontologies. New domain-specific ontologies can thus easily be defined by extending the core ontologies.

Second, the continuous care core ontology was developed in a modular way instead of as one big monolithic semantic model. The application of the participatory ontology engineering methodology resulted in seven core ontologies for the continuous care domain, namely the *Upper*, *Sensor & Observation*, *Context*, *Profile*, *Role&Competence*, *Medical* and *Task* ontologies. These modules are linked to each other using the OWL import mechanism. This way, the domain-specific ontologies can easily import and extend a specific core module instead of importing the whole continuous care core ontology. This facilitates re-use and allows application components to only use a subset of the continuous care core ontology to perform the domain-specific reasoning. A smaller, focused ontology is also easier to interpret and extend with new concepts, relations and definitions.

**Figure 2:**
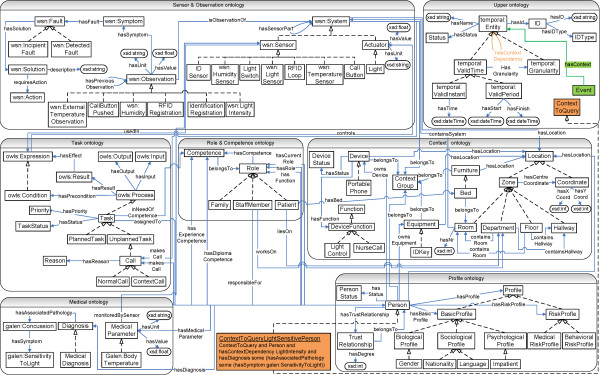
**Overview of the most prevalent classes and relations of the seven continuous care core ontologies.** The figure visualizes the most prevalent classes, their relations and properties of the seven continuous care care ontologies, namely the *Upper*, *Sensor*, *Context*, *Profile*, *Role & Competence*, *Medical* and *Task ontology*. The squares represent the classes. The dashed arrows depict subclass relationships. The blue arrows represent relationships between classes (object properties) and between classes and ranges (datatype properties). Ovals represent the ranges of the datatype properties.

As a final measure, the defined concepts are grouped as much as possible into logical categories, according to the properties they share. This logical category is introduced as a concept in the ontology and the grouped concepts are defined as subclasses of this concept. For example, consider the process of modeling the profile information of a person, e.g., his/her mother tongue, sex and nationality. This information could be represented by relationships, e.g., hasSex or hasNationality, with as domain the Person concept or as separate concepts in the ontology, e.g., Sex or Nationality. In the continuous care ontology the latter approach is chosen and these concepts are introduced as subclasses of a logical category concept, i.e., Profile. Restrictions, relations and properties can then be defined on this logical category. When new profile information needs to be added to the ontology, it can easily be added as a subclass of the logical category concept. The new profile information will automatically inherit all the relations, restrictions and properties defined on the parent Profile concept. This makes it easier to manage and extend the ontology. As can be seen at the bottom of Figure 2, the logical category can even be further divided in several logical subcategories, e.g., the subclassing of the Profile concept into the Biological, Psychological and SociologicalProfile concepts.The continuous care core ontologies are used by the SCB to filter the context information for the appropriate application components. The most important concepts and relations of the core ontologies, with respect to the use case detailed in the following section, are depicted in Figure 2 and discussed in the following paragraphs. The application components use (a subset of) the core and domain-specific ontologies to perform sophisticated reasoning on the data they receive from the SCB.

The *Upper ontology* describes general classes, relations and axioms. Most importantly this ontology enables data to be related with a unique ID. The classes preceded by the namespace prefix temporal are imported from the *SWRL Temporal Ontology*[64] and model complex interval-based temporal information. All the other core ontologies import this ontology and define their concepts as subconcepts of temporal:Entity. This is not shown on Figure 2 to avoid overloading the figure.

The *Sensor & Observation ontology* is one of the most important ontologies for filtering data. The concepts preceded by the wsn namespace prefix are imported from the *Wireless Sensor Network ontology* (WSN) [65, 66], which was developed by the co-authors and allows giving meaning to data values monitored by sensors. It contains a System concept, which models a system and its components. These components are modeled as subclasses of System, e.g., the Sensor or Actuator concepts. A System can then be associated with its components through various properties, e.g., hasSensorPart or hasActuatorPart. The modeled Observation concept represents a data value monitored by such systems. However, context information is often unreliable as it is gathered by sensors which can be imprecise or erroneous, e.g., fall detection sensors are known to often generate false positives. Moreover, the context information can be ambiguous as information gathered by different sensors can be conflicting or the context information might even be incomplete if there is no sensor information available. As it is mostly this context information that determines the behavior and the strategies of the different application components, it is important to make the quality of the context data explicit to prevent error propagation. To support the development of QoC-aware algorithms, this ontology contains axioms and rules, modeled as Symptom concepts, which allow detecting specific phenomena in the observations published to the SCB. For example, the LightIntensityBelowZeroSymptom detects light intensity observations that are below zero. Using OWL2 DL mechanisms, axioms are provided that reclassify these symptom individuals as individuals of the Fault concepts, e.g., the previously mentioned symptom is reclassified as a FaultyLightIntensitySensor indicating that the sensor that made the measurement is faulty, since light intensity can never be below zero. Additionally, a fault can be reclassified as a Solution, e.g., the previous fault is reclassified as the DoNotUseSensor solution indicating that measurements from this sensor should not be used by the algorithms. Consequently, the application components can take these classifications into account in their filter rules and algorithms. For example, on the one hand, the application components can register filter rules that indicate that observations reclassified as FaultyLightIntensitySensor concepts should not be forwarded to the application component. On the other hand, application components could also choose to receive these annotated observations and process them differently in their own algorithms, e.g., for Fault Detection and Diagnosis (FDD). The WSN ontology was extended with subclasses of the System, Sensor, Actuator, Observation, Fault and Solution concepts. These subclasses represent systems (e.g., nurse call system), sensors (e.g., RF or light sensor) and actuators (e.g., light or magnetic lock) and their associated observations (e.g., call button pushed or light intensity), faults (e.g., light intensity too high) and solutions (e.g., turn down the light) that play an important role in continuous care settings.

The *Context ontology* models the contextual environment information. ContextGroup is the most important concept. It represents a logical grouping of entities that belong together, e.g., a patient with all his/her devices, room, bed and other equipment. The composition of a context group dynamically changes based on the context. This ontology also contains all the information related to localization. A Location can either be a coordinate or a zone.

The *Profile Ontology* models the profile information about staff members and patients that was indicated as being important by the stakeholders in the workshops. Each Person is associated with a Profile, which consists of a basic and a risk profile. The latter is defined by axioms and rules, which allows inferencing the risk profile of the patient by reasoning on the information in the basic profile.

The *Role & Competence ontology* defines each role by its competences through axioms. This supports algorithms that find the most appropriate staff members to fulfill a task based on the required competences. Each person is then associated with competences and roles through five relationships: hasFunction, hasRole, hasCurrentRole, hasDiplomaCompetence and hasExperienceCompetence. The hasFunction relation models the primary role of a person, i.e., the role for which this person was primarily hired. The hasRole relation indicates all the roles a person can fulfill, while the hasCurrentRole models the person’s current role. If the latter is not instantiated, it is assumed that the current role of the person is his/her function. The hasDiplomaCompetence and hasExperienceCompetence indicate extra competences a person has acquired by either following training courses or through experience.

The *Galen Common Reference Model*[52], of which the concepts are preceded by the galen namespace prefix, represents clinical terminology. The *Medical ontology* adds axioms and constraints to this imported terminology, which express relations between this medical knowledge and concepts in the other ontologies.

Finally, the *Task ontology* models continuous care process workflows. A workflow represents a sequence of related continuous care tasks, which are conducted in a particular order. For this, the *OWL-S Process ontology*[67] was imported, of which the classes are preceded by the owls namespace prefix. The Process concept models a process, which can return information and produce a change in the environment based on the context and the information it is given. This is described by hasInput, hasOutput, hasPrecondition and hasEffect relations. A process can be composed of several other processes. The Task concept, introduced as subclass of Process, represents the various continuous care tasks. Consider, for example, the task of assigning a person to a call. A Call is modeled as an unplanned task. A Normal Call is then modeled as a Call, which has as precondition that a patient pushes a call button. This task takes the patient as input and the assigned caregivers as output. The effect of the Normal Call is that the assigned caregivers’ cellphones ring.

### Use case: optimizing continuous care through an ontology-based nurse call system

#### Scenario description

Nurse call systems are a very important fundamental technology in continuous care as they are used by caregivers to coordinate work, be alerted of patient’s needs, communicate with them through intercoms, and request help from other staff members. When patients feel unwell they push a button. The nurses then receive a message on a beeper with the room number. This brings us to the question: which nurse goes to the room, the closest one, the one on call, et cetera? The current systems often have a very static nature as call buttons have fixed locations, e.g., on the wall next to the bed. Moreover, there is an increased risk when patients become unwell inside a hallway, staircase or outside as they cannot use the nurse call system. Additionally, the current nurse call algorithms consist of predefined links of beeper numbers to rooms. Consequently, the system presently does not take into account the various factors specific to a given situation, such as the pathology of a patient (e.g., heart patient or confused) nor the competences of the staff (e.g., nurse or caregiver).

**Figure 3:**
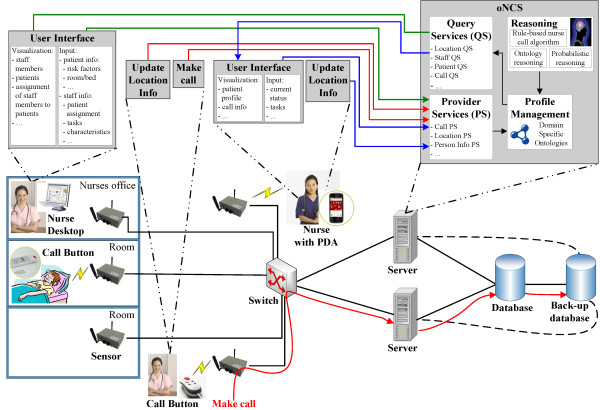
**General concept of the oNCS system with probabilistic priority assessment and profile management.**

The increased introduction of electronic devices in continuous care settings facilitated the development of the ontology-based Nurse Call System (oNCS), visualized in Figure 3, which allows patients to walk around freely and use wireless nurse call buttons. Additionally, this platform manages the profiles of staff members and patients in an ontology. A sophisticated nurse call algorithm was developed by the authors. It first determines the priority of the call using probabilistic reasoning algorithms, which take into account the origin of the call and the pathology of the patient. Next, the algorithm finds the most appropriate staff member to handle the call. It dynamically adapts to the situation at hand by taking into account the context, e.g., location of the staff members and patients. A detailed description of this platform can be found in Ongenae, et al. [68].

To better illustrate the benefits of the OCarePlatform, an extension of the oNCS Application Component is presented in this paper, which allows nurse calls to be automatically launched based on the data generated by the electronic equipment and sensors in the environment, e.g., alerting a nurse when the light intensity is too high in the room of a patient with a concussion. The proposed extension provides a solution to potential risky situations being missed because the caregivers are overloaded with constantly monitoring and orchestrating all the devices in the ambient patient room, making it more applicable to real-time scenarios.

To realize this extension, two other application components were designed, namely a Localization and Home Automation Application Component. The first determines the location of patients, staff members and devices. The latter automatically controls the ambient-intelligent activity in the room of the patient, e.g., switching on the lights at the appropriate level. The oNCS, the Home Automation and Localization Application Component each represent an *Application Component* which uses the SCB to filter context information that is relevant for it at that moment. The architecture of these components is thus very similar to the architecture of the *Application Component* at the top right of Figure 1. These three application components, the SCB, the sensors and the actuators together form a scenario-specific implementation of the OCarePlatform.

The OCarePlatform has also been leveraged to develop a social-aware and context-aware multi-sensor fall risk assessment and detection platform and to support the continuous care of elderly by a trusted care network in an ambient-aware home environment. More information about these use cases can be found in De Backere, et al. [69] and Van den Abeele, et al. [70] respectively.

#### Flexible and semantic publish/subscribe mechanism

The SCB allows application components to subscribe to relevant context information by registering filter rules with the Context Disseminator. When a context publisher forwards information to the SCB, the Context Disseminator reasons on the core ontologies to determine which subscribers are interested in this information by computing whether the forwarded data satisfies at least one filter rule defined by the subscriber. If it does, the information is forwarded to the application component.

**Figure 4:**
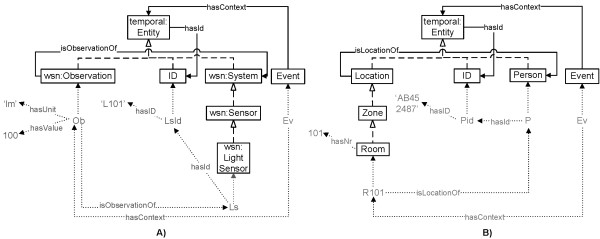
**Examples of events published on the SCB.** This instantiation diagram visualizes two examples of events that are published on the SCB, namely **A)** an event indicating that a light sensor with ID L101 measured a light intensity of 100 lumen and **B)** an event expressing that a person with ID AB452487 is located in room 101. The squares represent classes from the core ontologies. The dashed arrows depict subclass relationships. The blue arrows represent relationships between classes (object properties). The green text and arrows indicate instances of classes, ranges of datatype properties and instantiated object properties.

**Publishing context** The Context Provider Services are used to semantically annotate data with concepts from the core ontologies so that it can be interpreted by the SCB. To achieve a flexible system, in which published context and filter rules can easily be matched, an Event concept is added to the *Upper Ontology*, which has a hasContext relationship to temporal:Entity concepts. These additions are indicated in green in Figure 2. As all the other core ontologies import the *Upper Ontology*, this Event concept and the hasContext relationship essentially become a part of all the other core ontologies too. These core ontologies have defined all their concepts as subconcepts of temporal:Entity. As such, all their instances can occur as range of the hasContext relation. New types of events and filter rules can thus easily be expressed and matched. No other modifications to the ontologies are needed to support the management of these events.When a device wants to publish context information, the Context Provider Service creates an event, containing the data it wishes to publish. For example, to publish that the light sensor with ID L101 measured a light intensity of 100 lumen, the following instances are created (cf. Figure 4A):

- Ob **owl:instanceOf** Observation **and** Ob hasValue 100 **and** Ob hasUnit ‘lm’
- LsId **owl:instanceOf** ID **and** LsId hasID ‘L101’
- Ls **owl:instanceOf** LightSensor **and** Ls hasId LsId **and** Ob isObservationOf Ls
- Ev **owl:instanceOf** Event **and** Ev hasContext Ob

Similarly, application components can publish the results of their reasoning. For example, the Localization Component collects all the context information that gives an indication of the location of staff members, such as Radio-Frequency IDentification (RFID) tags, and calculates their current locations, e.g., room 101. These locations are published on the SCB to be used by other application components, e.g., the oNCS Application Component, as follows (cf. Figure 4B):

- Pid **owl:instanceOf** ID **and** Pid hasID ‘AB452487’
- P **owl:instanceOf** Person **and** P hasId Pid
- R101 **owl:instanceOf** Room **and** R101 hasNr 101 **and** R101 isLocationOf P
- Ev **owl:instanceOf** Event **and** Ev hasContext R101

To support the aggregation of context data and allow filter rules to process multiple observations simultaneously, more complex Context Provider Services can be written. For example, instead of publishing each light intensity observation to the SCB separately as in the first example, a Context Provider Service was developed to calculate the average of all the light intensity observations in a room within a minute and only publish this average as an event on the SCB. This event can be published as a normal LightIntensity observation or the aggregation process can be made explicit to the SCB by creating a new type of the Observation concept in the *Sensor & Observation core ontology*, for example AveragedLightIntensityObservation. Filter rules can then be written to process this new type of event. Similarly, Context Provider Services can be written to aggregate the values of different types of sensors.

**Subscribing to context** Filter rules are expressed by defining subclasses of the Event class. This way, the Context Disseminator can determine if the published context matches a filter rule by asking an OWL Reasoner, such as Pellet, if the published event can be classified as an instance of the Event defined by the filter rule [59]. The filter rule is defined by means of necessary and sufficient conditions, which the published event must fulfill to belong to this class. Moreover, the Context Disseminator can use the OWL reasoner to check if the filter rule is satisfiable, i.e., does not contradict with the knowledge already defined in the core ontologies. If the filter rule is unsatisfiable, the subscription of the filter rule fails and the class is not added to the ontology. This ensures increased robustness.

For example, as the Home Automation Component regulates the ambient-intelligent activity in the room of a patient, it is interested in location information, as well as light intensity, humidity and temperature observations. It registers the following filter rule:

Event **and** hasContext **some** (LightIntensity

**or** ExternalTemperatureObservation

**or** Humidity

**or** Location)

Note that the two Event examples match with this filter rule. The ontology declares that each Observation, which has a unit of type lumen, is an observation of type LightIntensity, as follows: LightIntensity == (Observation and (hasUnit value “lm”^ˆˆ^ string)) Consequently, the first event matches. Similarly, the second event matches because the ontology states that each Room is a subclass of Location, as follows: Room subclass of Zone Zone subclass of Location These events will thus be forwarded to the Home Automation Component.

**Generating new filter rules** The information in which an application component is interested can change based on the current context. Instead of filtering all the context that might be needed at some point in time, the application components are able to generate new filter rules when the context changes. The filter rule generation process is thus made context dependent. To enable this, the ontology allows defining context dependencies between different, but related context. A context dependency (X,c,Y) means that context Y only needs to be filtered if the condition c holds for context X. Optionally, a range d can also be specified for the values of this context parameter Y. For example, the domain-specific ontology used by the oNCS Application Component defines the dependency: (Person, hasDiagnosissome(hasAssociatedPathologysome(hasSymptomsomegalen:SensitivityToLight)), LightIntensity). This dependency indicates that the oNCS Application Component is also interested in LightIntensity measurements when a patient is detected who is sensitive to light. Additionally, the domain-specific ontology associates each light sensitivity symptom with a threshold, e.g., 100 lm. Consequently, the range d of Y is defined as being greater than or equal to this threshold. This allows the oNCS Application Component to alert a caregiver when the light intensity is too high, i.e., greater than or equal to 100, at the location of this patient.

Given the context dependencies, the filter rule generation algorithm works as follows. The ContextToQuery concept and the hasContextDependency relationship between temporal:Entity concepts are introduced in the *Upper Ontology*, as visualized in orange in Figure 2. To define a context dependency, a subclass of the ContextToQuery concept is defined with the necessary and sufficient condition that the ontological instance must have the relationship X hasContextDependency Y and *c*≡*true*. The ContextToQueryLightSensitivePerson concept at the bottom of Figure 2 illustrates this for the running light sensitivity example. Every time the context changes, the context dependencies are investigated by examining the membership of instances to the ContextToQuery concept through inferencing on the ontology. Once the membership is determined, the construction of the filter rules is straightforward. For example, consider that the oNCS Application Component is alerted that the patient in room 101 is diagnosed with a concussion, because his profile is updated in the EHR database. The domain-specific ontology of the oNCS Application Component contains the knowledge that patients with a concussion have light sensitivity as a possible symptom, as follows: Concussion subclass of (hasSymptom some LightSensitivity) Consequently, the condition c of the context dependency holds and the reasoning inferences that this person is an instance of the ContextToQueryLightSensitivePerson concept.

The filter rule is then constructed by defining an Event, which has as context the range Y of the hasContextDependency. To determine the device(s) from which this context Y should be filtered, the ContextGroup associated with the patient is analyzed. As previously explained, the ContextGroup represents a logical group of entities, e.g., a patient with his/her associated devices. For the running example, this results in the following filter rule:

Event **and** hasContext **some** (LightIntensity

**and** (hasValue **some** float[ >=100])

**and** (isObservationOf **some**

(hasId **some** hasID ‘L101’)))

This rule filters light intensity observations from the room of the patient. For the published context to match with this filter rule, it must be of type LightIntensity. Moreover, its current value must be of type float and higher than or equal to 100. Finally, the observation must originate from a system with ID ‘L101’. This is the ID of the light sensor in room 101. As previously indicated, this kind of static information is stored in databases, which can be queried by the application components. Note that the first example of the “Publishing context” Section matches with this filter rule. More information about this filter rule generation algorithm can be found in [59].

**Figure 5:**
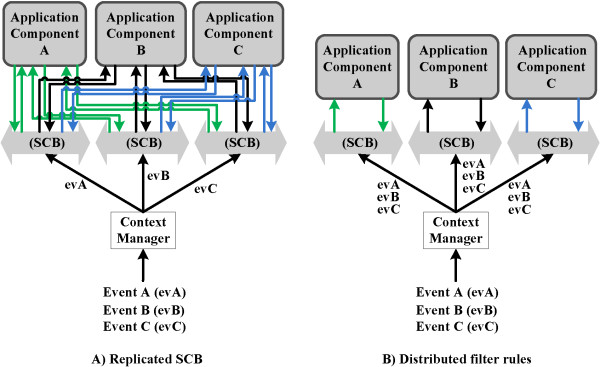
**Comparison of the two approaches to distribute the SCB.** To prevent that the SCB becomes a single point of failure and to improve the performance, the SCB can be distributed. Approach **A** replicates the SCB, meaning that each context dissemination instance will contain the same amount of filter rules and the published events are divided across the different SCBs and processed in parallel. Approach **B** distributes the filter rules across different instances of the SCB, processing a published event in parallel.

**Distributed deployment** As the application components heavily depend on the SCB to receive relevant context information, the centralized design of the SCB forms a single point of failure and a performance bottleneck. However, the SCB can easily be distributed as it does not retain the published context data, i.e., only one event is processed at a time by the SCB. Different instances of the SCB, processing the various events in parallel, can thus easily be deployed without interfering with each other. An actual large-scale deployment of the SCB will thus most likely not correspond to a centralized physical process, but will be a virtual substrate distributed across the network. Two approaches can be used, which are compared in Figure 5.

On the one hand, the SCB can be replicated, meaning that each context dissemination instance will contain the same amount of filter rules and the published events are divided across the different SCBs and processed in parallel. This improves the scalability. Replication also removes the single point of failure as a replicated instance of the SCB can easily replace a faulty one. This approach can be achieved by using the persistent team approach described in the Adaptive Agent Architecture [71].

On the other hand, the filter rules can be distributed across different instances of the SCB, processing a published event in parallel. In this case, the number of filter rules present in each context dissemination instance will thus be relatively low, which increases the throughput of each SCB significantly. Sophisticated algorithms have been proposed in literature to efficiently distribute the filter rules across the different peers [72]. This approach can be combined with the first to deal with faulty nodes.

### Evaluation set-up

**Figure 6:**
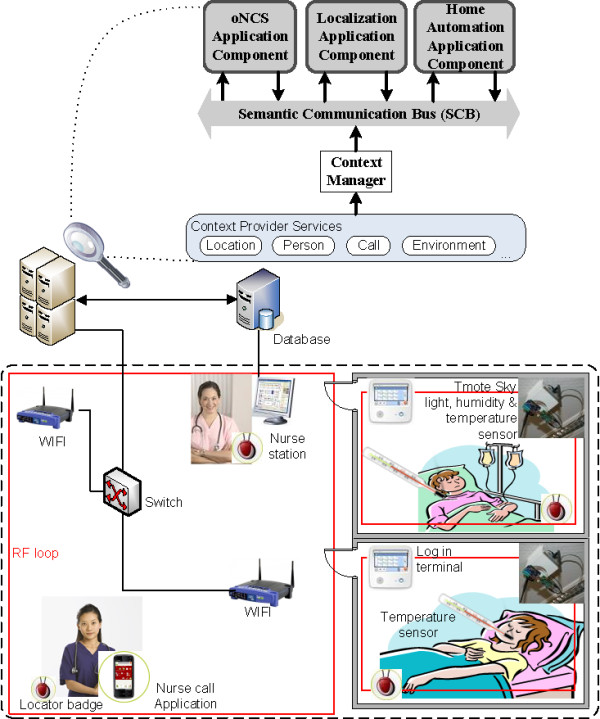
**Deployment of the prototype in PRoF.** To evaluate the performance and benefits of the SCB, a prototype of the oNCS Application Component, extended with the Localization and Home Automation Application Component, was deployed in the Patient Room of the Future (PRoF). PRoF consists of a typical patient room and hallway found in a hospital setting, as well as a room mimicking a homecare setting. For the prototype, both rooms were equipped with a TMote Sky sensor board, which contains a light, temperature and humidity sensor. Also, each patient and staff member carried an RFID tag to track his/her location. Finally, each patient wore a bracelet that monitors the patient’s body temperature. The developed prototype, consisting of the various context providers, the SCB, the oNCS, the localization and Home Automation Application Components, was installed in PRoF and integrated with the available nurse call and light control system, RF tags and sensors. Smartphones running the mobile nurse call application, which enable the nurses receive and answer calls, were also provided.

To evaluate the performance and benefits of the SCB, a prototype of the oNCS Application Component, extended with the Localization and Home Automation Application Components, was implemented and tested in the Patient Room of the Future (PRoF) [73]. PRoF is a high-fidelity mock-up of a near-future patient room integrating innovations from soft- and hardware developers as well as furniture. The aim of PRoF is to make a patient feel more at home by exploring ways to put the patient and his needs first. PRoF consists of a typical patient room and hallway found in a hospital setting, as well as a room mimicking a homecare setting. For the prototype, both rooms were equipped with a TMote Sky [74] sensor board, which contains a light, temperature and humidity sensor. Also, each patient and staff member carried an RFID tag to track their location. Finally, each patient wore a bracelet that monitors the patient’s body temperature. The developed prototype, consisting of the various context providers, the SCB, the oNCS, and the localization and Home Automation Application Components, was installed in PRoF and integrated with the available nurse call and light control system, RF tags and sensors. Smartphones running the mobile nurse call application, which enable the nurses receive and answer calls, were also provided. Figure 6 visualizes the deployment of the prototype and accompanying sensors in PRoF. As PRoF contains only two rooms and the number of users was also limited, only one instance of the SCB was deployed.

This prototype allowed users to experience a fully immersed, contextual experience of the system in a lifelike context. As we wanted the participants to have a complete experience of the system, small groups were invited, i.e., two or three users per workshop, so that they would be occupied at all times and the researchers could follow them one-on-one. As such, seven workshops were organized for 15 participants. During a 2.5 hour role-playing workshop, the participants were asked to play out seven scenes. For each scene, a test user received a persona card and a context card, informing the test user of the role he or she would have to take up and what he or she would have to do. Afterward, the functionality of the system was elaborately discussed.

However, as PRoF contains only two rooms, it was difficult to thoroughly evaluate the performance of the system based on these user tests. To mediate this, simulations were performed based on realistic data gathered from observations and interviews performed at Ghent University Hospital [75]. The simulated department contains 20 rooms, 30 patients and 10 staff members, who answer calls. It was again assumed that each room is equipped with a TMote Sky, the temperature of each patient is monitored with a bracelet and the location of all the staff members and patients is tracked. The simulation of the observations of the RF tags was based on realistic data gathered about the walking behavior of caregivers and patients in several departments of Ghent University Hospital. The simulation of the observations of the other sensors was based on stakeholder input. Table 2 gives an overview of the amount of data generated by each sensor for the simulations. As the goal of the simulations was to assess the performance and scalability of the SCB, only one instance was deployed.

**Table 2:** **Sensor data generated in a department with 20 rooms, 30 patients and 10 staff members**

	Nr. of	Nr. of observations	Total nr. of
Sensor	sensors	per sensor	observations per hour
Light	1/room	1/sec	72,000
Temperature	1/room	1/sec	72,000
Humidity	1/room	1/sec	72,000
RFID tag	1/person	1/sec	144,000
Body temperature	1/patient	1/sec	108,000

**Figure 7:**
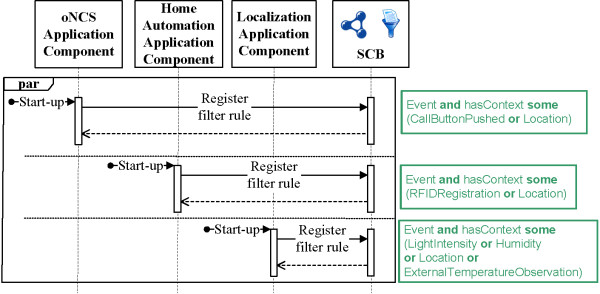
**First step of the implemented scenario: Configuration.** This sequence diagram visualizes the different actions taken during the first step of the scenario realized by the implemented prototype. In this step, the different application components are configured by registering filter rules with the SCB such that they receive the context information that they are always interested in, independent of the current context. The filter rules are depicted in green. The “par” indicates that the different application components can perform their actions in parallel.

The prototype is able to realize the example detailed in the “Introduction” Section. This scenario consists of the following steps.First, the oNCS, Localization Component and Home Automation Application Components register filter rules with the SCB, to receive context information they are interested in, independent of the current context. These filter rules are visualized in green in Figure 7.

**Figure 8:**
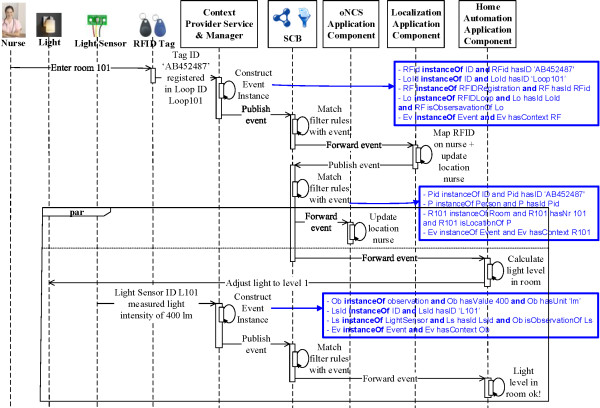
**Second step of the implemented scenario: Turn on light when nurse enters the room.** This sequence diagram visualizes the different actions taken during the second step of the scenario realized by the implemented prototype. In this step, a nurse enters the room of the patient. This is detected by using RFID Tags, which send an *Event* to the SCB. The Localization Component is alerted of this *Event* as it matches with the filter rule this application component registered with the SCB. The location of the nurse is updated and this information is published on the SCB. This location information is forwarded to the oNCS and the Home Automation Application Components as it matches with their filter rules. The first just updates the location of the nurse in its local domain-specific ontology. The second uses this information to adjust the light level in the room of the patient. Finally, the light sensor in the room notices the change in light intensity and publishes this *Event* on the SCB. This *Event* matches with the filter rule of the Home Automation Component, which receives this *Event* and reasons that the light was adjusted to the appropriate level. The different Events are depicted in blue. The “par” indicates that the different application components can perform these actions in parallel.

**Figure 9:**
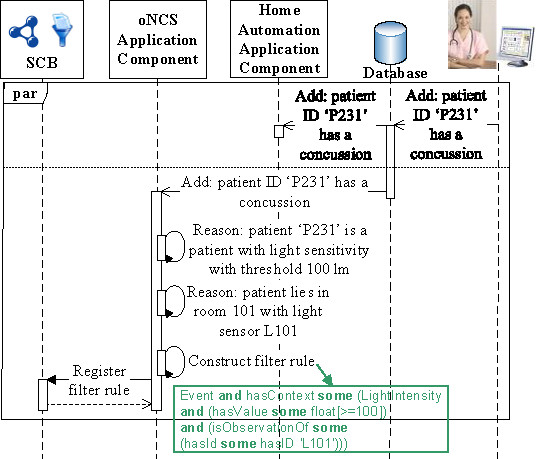
**Third step of the implemented scenario: Registering a dynamic filter rule.** This sequence diagram visualizes the different actions taken during the third step of the scenario realized by the implemented prototype. In this step, the local domain-specific ontologies of the Home Automation and oNCS Application Components are updated to signal to these components that a particular patient has a concussion. The oNCS Application Component uses its local domain-specific knowledge to derive that this patient is sensitive to light. Consequently, the oNCS Application Component registers a filter rule to receive specific context information pertaining to the light intensity in this patient’s room. The filter rule is depicted in green. The “par” indicates that the different application components can perform their actions in parallel.

**Figure 10:**
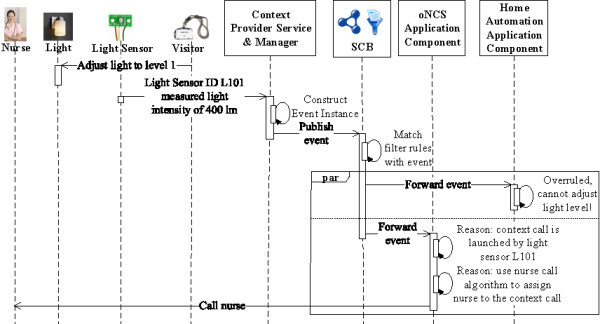
**Fourth step of the implemented scenario: Visitor overrules the system and a nurse is alerted.** This sequence diagram visualizes the different actions taken during the fourth step of the scenario realized by the implemented prototype. In this step, a visitor adjusts the light level in the room and thus overrules the system. The light sensor in the room notices the change in light intensity and publishes this *Event* on the SCB. This *Event* matches with the filter rule of the Home Automation Component, which receives this *Event* and reasons that the light level cannot be adjusted to the needs of the patient as the component has been overruled. However, this *Event* also matches with the new filter rule that the oNCS Application Component registered in step 3 of this scenario. The oNCS Application Component derives that the light level is above the threshold for patients with a concussion and uses reasoning to find the most appropriate nurse to alert. The “par” indicates that the different application components can perform these actions in parallel.

During the night, a nurse enters the room of a patient, who is sleeping. As the RFID tag of the nurse is picked up by the loop installed in the room, an event is generated and published on the SCB. This event is shown in the first blue square of Figure 8. The RFIDRegistration concept is defined in the ontology as an observation made by a Sensor of type RFIDLoop or RFIDReader, as follows: RFIDRegistration == (Observation and (isObservationOf some (RFIDLoop or RFIDReader))) Consequently, this event matches with the filter rule registered by the Localization Application Component. This component maps the RFID on the appropriate nurse and updates the location of this person. This location information is pushed back to the SCB, as illustrated in the second blue square of Figure 8. As already explained in the “Subscribing to context” Subsection of the “Flexible and semantic publish/subscribe mechanism” Section, this location information matches with the filter rule of the Home Automation Application Component. Similarly, it also matches with the filter rule of the oNCS Application Component. The latter only updates the location of the nurse. No further reasoning is triggered in this component. However, the former detects that a nurse has entered the room during the night shift. The Home Automation Application Component also knows that the light is currently turned off. To enable the nurse to perform her duties, the component decides to switch on the light to a very low level, namely 1. Consequently, the light sensor in the room detects the changed light intensity in the room and an event is sent to the SCB, as shown in the third blue square of Figure 8. As explained in the “Subscribing to context” Subsection, this light intensity information is picked up by the Home Automation Application Component, which concludes that the light level was adjusted to the right level.The next day, the EHR of the patient is updated to indicate that a patient with ID P231 has a concussion, as illustrated in Figure 9. Consequently, the knowledge bases of the oNCS and Home Automation application components is updated with this information as they are regularly synched with the database containing the EHR data. As explained in the “Generating new filter rules” Subsection of the “Flexible and semantic publish/subscribe mechanism” Section, the oNCS Application Component registers a new filter rule with the SCB as it detected that the patient with ID P231 is sensitive to light.In the evening, a visitor enters the room of patient with ID P231, who is currently resting. This causes the light to be automatically turned on to a low level. The actions taken to realize this are similar to the ones visualized in Figure 8. However, in Figure 8 the Home Automation Application Component dims the light because it is night. In this case, the light is dimmed because the patient has a concussion. However, as visualized in Figure 10, it remains possible for people to overrule the system and brighten the light in the room. The light sensor again picks up this change in light intensity in the room. An event similar to the event in the last blue square of Figure 8 is published on the SCB. This event is picked up by the Home Automation Application Component. However, this component detects that it already tried to dim the light and that a person has manually adapted the light level. The Home Automation Application Component can thus not intervene. However, because of the newly registered filter rule, this event is also forwarded to the oNCS Application Component. This component reasons on the event and alerts a nurse that the light level has been turned on too high in the room of a patient with light sensitivity.

The evaluations were performed using the continuous care core ontologies needed to model the scenario as described in the “Continuous care ontology” Subsection. The core ontologies consist of 142 classes, 42 object properties, 21 data properties and 556 asserted axioms. The Protégé ontology editor [76, 77] was used to develop the ontologies in OWL-DL [20]. The context information published on the SCB and the filter rules registered by the application components are also expressed in OWL-DL. The prototype was built in Java, based on the Pellet OWL 2 reasoner [22] and the OWL Application Programming Interface (OWL-API) [78]. The cache was implemented using a combination of the Least-Frequently Used (LFU) and Least Recently Used (LRU) replacement algorithms, called the Least Recently/Frequently Used (LRFU) policy [79]. All the tests were performed under exactly the same conditions on the same isolated machine with following specifications: Advanced Micro Devices (AMD) Athlon 64 X 2 Dual Core Processor, 3000 megahertz (MHz) Central Processing Unit (CPU) and 2 Gigabyte (GB) of Random-Access Memory (RAM).

## Results and discussion

Semantic reasoning and ontologies were adopted in this research to facilitate the exploitation and integration of heterogeneous context information delivered by the devices in an ambient-intelligent patient room. However, it is widely known that special attention needs to be paid to the number of instances in the ontology as this adversely affects the reasoning performance [80]. Consequently, if all the data from the ambient-intelligent environment would be delivered directly to the ontology-based application components, their performance would degrade drastically. By using the SCB, which only process one event at a time, filters can be defined to ensure that the application components only receive relevant context information. This way, the different infrastructure components are loosely coupled, effectively separating the devices delivering context information from the application components that process this information. Moreover, the SCB allows the composition of complex application components from a set of smaller application components, which perform specific reasoning tasks in parallel and forward their conclusions through the SCB.

Consequently, the effectiveness of the rules can be measured by the reduction in the amount of information that is processed by the application components as it is important that only the necessary context information is analyzed. However, the correctness of the rules is another important performance metric. The correctness is influenced by the amount of data that was wrongfully filtered. It is important not to filter too much information, as a lack of information about the context might cause application components to take incorrect actions or no action at all. The goal is to increase the effectiveness, while maintaining the correctness. Consequently, we aim to maximize the amount of data that is not forwarded, while ensuring that all the information that influences the actions of application components is correctly forwarded.

In the scenario detailed in the “Evaluation set-up” Subsection of the “Methods” Section, the SCB is capable of filtering a large amount of the simulated data from Table 2. As the Home Automation Component is only interested in sensor observations about the light intensity, the external temperature and the humidity, 54% of the generated sensor data is not forwarded to this component. Similarly, the Localization Component is only interested in RFID tag data, resulting in a 77% reduction of forwarded data. By taking advantage of the dynamic filter rule generation, none of the light intensity observations are forwarded to the oNCS Application Component if none of the patients in the department have light sensitivity symptoms. When the department does have such a patient, only 8.3% of all the light intensity observations are forwarded to the oNCS Application Component. In this case we assume that it takes the nurse on average 5 minutes to respond to the context call, generated by the oNCS Application Component because the light intensity is too high in the room. This time interval was chosen based on observations and stakeholder expertise. This way, the filter rule generation process allows dynamically adapting the amount of data that is filtered based on the context. It can be noted that neither the oNCS nor the Home Automation Application Component ever receive RFID tag observations anymore. They depend on the Localization Application Component, which processes these raw RFID observations and publishes the resulting augmented location information. However, these location updates are far less frequent than the RFID tag observations, as only significantly changed locations are published, e.g., staff member is in another room.

**Figure 11:**
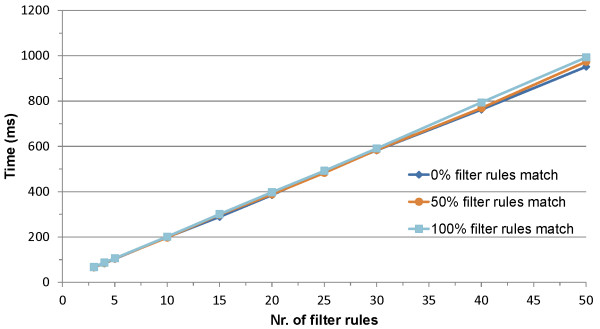
**Average reasoning time as a function of the number of filter rules.** This graph visualizes the average reasoning time (y-axis in milliseconds (ms)) needed to publish, filter and forward one event as a function of the number of filter rules (x-axis) and the percentage of filter rules that match with this event, averaged over 30 iterations in case a cache miss occurs. The dark blue, orange and light blue line indicate that 0%, 50% and 100% of the filter rules match with the published event respectively.

The dissemination of new context consists of two steps. First, filter rules are created by the application components and registered with the Context Disseminator of the SCB. However, as filter rule registrations only occur occasionally, the introduced delay is negligible. Second, context is published to the SCB and matched with the filter rules. If a match is found, the context is forwarded to the appropriate application component. The publication of context information happens frequently, as illustrated by Table 2. As such, it is important that events are matched with filter rules quickly and efficiently.When a cache miss occurs, the Context Disseminator has to reason on the information in the ontology to match the published event to the registered filter rules. The performance of filtering, matching and forwarding an event with the SCB as a function of the number of filter rules in case of a cache miss, is visualized in Figure 11. The lower and upper limits of the standard deviation are [2.19, 10.28], [2.58, 9.76] and [2.65, 10.07] when respectively 0%, 50% and 100% of the filter rules match with the published event. The graph shows that the processing time is linear in terms of the amount of filter rules and that the influence of the percentage of filter rules that match the event is negligible. Note that for the described scenario, which contains at most 4 filter rules, an event is processed in on average 82.67 ms. However, the performance quickly decreases. For 50 filter rules, it takes on average 1 second to process an event if a cache miss occurs.

**Figure 12:**
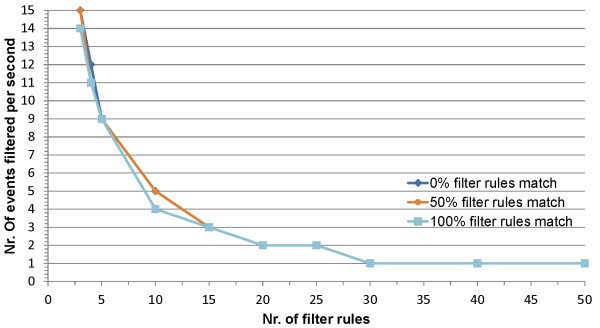
**Number of events processed per second as a function of the number of filter rules.** This graph visualizes the number of events that can be filtered per second (y-axis) by one instance of the SCB as a function of the number of filter rules (x-axis) in case none of these events are present in the ache. This means that the Context Disseminator will have to reason on the ontology to filter, match and forward these events to the correct application components. An event is only counted when it has been completely processed and forwarded. The dark blue, orange and light blue line indicate that 0%, 50% and 100% of the filter rules match with the published events respectively.

It can be derived from Table 2, that every second all the sensors will output a new observation. In order to keep up with the publishing rate of the sensors (throughput), it is thus important that the SCB can process one event of each sensor in less than a second. Figure 12 visualizes the number of events that can be filtered per second by one instance of the SCB as a function of the number of filter rules in case none of these events are present in the cache. This means that the Context Disseminator will have to reason on the ontology to filter, match and forward these events to the correct application components. Note that the number of events on the y-axis is limited to those events which have been completely processed within the time limit, i.e., 1 second. Partially processed events are not considered. The graph shows that for the described scenario, which contains at most 4 filter rules, at most 15 events can be processed. If this result is mapped on the sensors in Table 2, this means that the SCB can process the events of 11 sensors of the simulated department, namely the events of 1 staff member (carrying an RFID tag) and 2 rooms (each fitted with 1 light, 1 temperature and 1 humidity sensor) with 2 patients in each room (each carrying an RFID tag and a body temperature sensor).

To enhance these performance results, three measures can be taken. First, the cache can be used. If the published event is present in the cache, a cache hit occurs and no reasoning needs to be performed to process the event and forward it to the appropriate application components. As the cache is implemented using LRFU, looking up whether an event is present in the cache and, in case of a cache hit, getting the filter rules and thus the application components to which the event should be forwarded can be performed with a worst-case performance of O(log n), where n is the number of entries in the cache [79]. Moreover, replacing an entry in the cache with a new entry in case of a cache miss also has a worst-case performance of O(log n). Consequently, looking up an entry in the cache performs much better than the reasoning that needs to be performed by the SCB when a cache miss occurs. It needs to be noted that the entries in the cache will become invalid when a new filter rule is added to the SCB as it is possible that the events already present in the cache now also match on this new filter rule. To resolve this issue, all the entries in the cache are flagged. When a cache hit occurs for a flagged entry, the Context Disseminator will perform the reasoning step, update the value in the cache and remove the flag. This way the whole cache will eventually be updated, either by performing reasoning or because flagged entries are replaced by new entries. To further speed up the runtime performance of the SCB, the cache could be filled with representative events during start-up. For example, the cache could be filled with room temperature events between 18 and 22° Celsius, as these are the most common room temperature values. However, this will significantly increase the start-up time of the platform and might only be a good choice when the filter rules do not changeoften.

As a second measure, the framework can be distributed. As explained in the “Flexible and semantic publish/subscribe mechanism” Section, two approaches can be used, namely replicating the SCB or distributing the filter rules across multiple instances of the SCB. A combination of both can also be used to ensure scalability as well as tolerance against faulty SCB instances. The choice of the distribution approach depends on the specific use case scenario. For use cases where the number of published events is high compared to the number of filter rules, the first approach is more suitable. As illustrated by Table 2, the use case presented in this paper falls into this category. A lot of events are published on the SCB, but only 4 filter rules are registered. The first approach is thus a good choice. As mentioned previously, this can be achieved by using the persistent team approach. The only parameter of this approach, is the minimum number of times R that the SCB should be replicated. To achieve a scalable deployment for the use case under scrutiny, R is set equal to the number of rooms, i.e., 20. As an SCB instance with 4 filter rules is able to process 15 events per second, the availability of at least 20 SCB instances guarantees that the 3 environment observations measured by the TMote Sky and the 4 patient observations, namely the location and temperature of the two patients, in each room per second can be efficiently processed. It also leaves enough processing power to process the location observations of the staff members and to allow the registration of additional context-dependent filter rules. In contrast, the second approach or the combination of both approaches is a better choice for use case scenarios with a lot of filter rules compared to the number of published events. Next to the parameter R, specifying the number of SCB instances that should be created, this approach also requires a specification of how the filter rules should be distributed across these instances. A simple algorithm consists of distributing the filter rules so that each instance of the SCB contains approximately the same amount of filter rules. This ensures that each instance of the SCB has a comparableperformance.

Furthermore, it has been shown by the authors [59] that the performance of the SCB depends significantly on the complexity of the used core ontologies. It is therefore important to find a good balance between the desired throughput and the intelligence of the SCB, i.e., the expressiveness of the core ontologies and accompanying filter rules.

By balancing the expressivity of the core ontologies and the desired throughput, employing caches and distributing the SCB according to the specifics of the use case, the developed platform supports the realization of a plethora of healthcare scenarios in an efficient and scalable way.

To ensure the correctness of the filter rules it is important to continuously evaluate the platform with the domain experts, both during the development process and after the system has been deployed. Domain experts were constantly involved during the design and development of the continuous care ontologies, OCarePlatform and used application components to make sure the system correctly reflects the continuous work processes of the caregivers. Observations were performed to investigate which information is taken into account to perform a certain task or make a decision. A participatory methodology was used to develop the ontology and the accompanying algorithms. Moreover, the developed system has been thoroughly evaluated with the various stakeholders by allowing them to play scenarios in PRoF. If the system gets deployed, techniques can also be used to continuously monitor and improve the correctness. For example, situations can be examined in which the decision of the platform was overruled by the users and intermediate feedback can be gathered.

The user tests resulted in a considerable amount of feedback. However, the feedback mostly pertained to the user interface of the mobile nurse call application and the employed nurse call algorithm. No comments were made about missed or unnecessary calls, wrong light levels or the performance of the system. The ambient intelligence of the room, e.g., adjusting the lights or the temperature, was almost never overruled. Similarly, the calls that are automatically launched based on the context, were found interesting most of the time. However, the nurse that is assigned to the call was sometimes changed or overruled. It is sometimes difficult to assess whether a nurse is available to address a particular call, because it is difficult to assess what their current task is. The nurse call algorithm was then adapted to deal with this issue, by allowing caregivers to indicate that the call should be redirected. The system then finds another suitable nurse based on the current context information. This algorithm is more thoroughly described in Ongenae et al. [63].

Note that the described application components of the OCarePlatform only adjust the ambient intelligent devices, e.g., lights or radiators, or alert the caregiver of abnormal situations or measurements. The EHR of the patient is not adjusted by the components. A lot of user resistance was met in the fact that the system would automatically feed or adapt the EHR of the patient. The caregivers felt that human assessment is always needed of the alerts to assess their validity, as it is difficult for the system to capture all the small nuances of a situation that the caregivers are sensitive to because of their experience and everyday interaction with the patients. Therefore, it was chosen to leave the responsibility in the hands of the caregivers to assess whether the system made the right conclusion. This feedback about the correctness of the alert can be inserted by the caregivers on the mobile application. The caregiver can then choose to add the alerted information to the clinical record of the patient him- or herself. The latter could be easily automated by providing the caregiver with a button on the mobile applications that allows inserting the alerted information to the EHR. As we already map the EHR to the ontology, implementing the fact that information derived by the ontology should be inserted in the EHR is straightforward.

Future work on the continuous care ontology will focus on two things. First, it will be investigated how the ontology can be aligned with the Semantic Sensor Network Ontology (SSN) [81], which was constructed by the Semantic Sensor Network W3C Incubator Group [82]. Second, the ontology will be extended with new parts to support specific continuous care application domains, e.g., fall detection or activity recognition. Future work on the OCarePlatform will concentrate on providing tools and methodologies that allow developers to easily develop new services or adapt the algorithms in already existing services. Techniques will also be researched to combine the event-driven OCarePlatform with more request-driven workflow composition and execution platforms.

## Conclusions

In this article, a context-aware and pervasive framework was presented, capable of disseminating and filtering important care-related data of the different technologies available in an ambient-aware patient room towards a multitude of care applications, based on their information requirements. To realize this goal, the framework employs continuous care ontologies, which capture the information and knowledge being exchanged and utilized within healthcare settings. The applications can register, adapt and remove semantic filter rules on the fly to receive context information that is important to them at that moment. This way, the amount of data which needs to be processed by the applications is significantly reduced, which improves their performance and decreases overhead while maintaining an individualized approach.

It was shown that the delay introduced by the context dissemination and filtering component is linear in the amount of filter rules and is negligible when 10 or less filter rules are registered. The performance of the platform can be significantly increased by employing a cache and by distributing the reasoning on the filter rules. The latter is achieved by replicating the context dissemination and filtering component or by distributing the filter rules across different instances of this component. A combination of both approaches can also be used. Moreover, the platform supports the composition of complex applications from a set of smaller applications in a loosely coupled manner. The simple applications perform specific reasoning tasks in parallel and notify their conclusions to other applications, which have expressed an interest in this kind of information.

## Abbreviations

ACCIO: Ambient-aware provisioning of Continuous Care for Intra-muros Organizations
AMD: Advanced micro devices
CMF: Context managing framework
CMSANS: Context Management service with an awareness and notification service
CoBrA: Context broker architecture
CPU: Central processing Unit
DL: Description logics
EHR: Electronic health record
EPR: Electronic patient record
ERMHAN: Emilia Romagna mobile health assistance network
FDD: Fault detection and diagnosis
GB: Gigabyte
LFU: Least-frequently used
LRFU: Least recently/frequently used
LRU: Least recently used
MHz: Megahertz
OCarePlatform: Ontology-based care platform
oNCS: Ontology-based nurse call system
OWL: Ontology web language
OWL-API: Ontology web language application programming interface
PRoF: Patient room of the future
QoC-aware: Quality of context-aware
RAM: Random-access memory
RDF: Resource description framework
RFID: Radio-frequency iDentification
SCB: Semantic communication bus
SOA: Service-oriented architecture
SOCAM: Serive-oriented context-aware middleware
SWRL: Semantic web rule language
WSN: Wireless sensor network.
